# Trials trail a long tail

**DOI:** 10.1186/1745-6215-16-S2-P159

**Published:** 2015-11-16

**Authors:** Mike Clarke

**Affiliations:** 1 Northern Ireland Network for Trials Methodology Research, Belfast, UK

## Background

Systematic reviews are an important conduit for the findings of clinical trials. The Cochrane Database of Systematic Reviews (CDSR) is the world's largest collection of full-text reviews of interventions and provides an opportunity to investigate this. However, access data often focuses on the top 20 or 50 reviews, revealing nothing about several thousand more. Therefore, it might be assumed that a small set of reviews account for a large proportion of the accesses. This study examines this to see if CDSR has a “long tail” for access. This is the first investigation of this concept for such a large collection of health research sharing the same design.

## Objective

To determine if most access to Cochrane Reviews is to a small or large subset. Methods: The numbers of times that the full text for each Cochrane Review in the Cochrane Library was accessed during 2014 were provided by Wiley-Blackwell. (Data were not available from other platforms.) The 5584 reviews available throughout the year were ordered by number of accesses.

## Results

Cochrane Reviews have a long tail. The top 50 account for 11% of accesses, the top 1000 for 56%. 80% of accesses are reached at review 2263, and 98% at review 4570, when reviews still had > 10 accesses/month.

## Conclusion

This study of the length of the tail that trails for trials in systematic reviews is the first such exploration. It shows that users are accessing a much larger proportion of reviews, and hence their included trials, than others might think.

